# 

**Published:** 1887-06

**Authors:** 


					﻿JMtr* at (f,f M C, aiA^Hn.	MwO-dcw Matt Matter,
Vol. IL
JUNE, 1887.
No. 12
%1»* T W *F-« * <**
£ 8	1% I	I	L-	I	• Sh, *■■
* Q &	/* w 4 <Ls	»	«C	s	•••..
■i -. ^ y i>i
,«RJI
JOURNAL
F. E DANIEL., M. D., Editor and Publisher, AUSTIN TEXAS.
Sorthrrn Office, WI7 FJlbert Mtreet, Philadelphia.
Edgknk Von Boeckmann, Steam Book ano Jois Pbiwter, aTwtin. "oxa...
				

